# The role of diet in the pathophysiology and management of irritable bowel syndrome

**DOI:** 10.1007/s12664-020-01144-6

**Published:** 2021-03-05

**Authors:** Magdy El-Salhy, Tanisa Patcharatrakul, Sutep Gonlachanvit

**Affiliations:** 1 Section for Gastroenterology, Department of Medicine, Stord Helse-Fonna Hospital, Stord, Norway; 2 Department of Clinical Medicine, University of Bergen, Bergen, Norway; 3 Center of Excellence on Neurogastroenterology and Motility, Faculty of Medicine, Chulalongkorn University, Bangkok, Thailand; 4 Division of Gastroenterology, Department of Medicine, King Chulalongkorn Memorial Hospital, Thai Red Cross Society, Bangkok, Thailand

**Keywords:** Cellophane noodles, Chili, Enteroendocrine cells, Fecal microbiota transplantation, Fibers, FODMAP, Intestinal microbiota, NICE-modified diet, Rice

## Abstract

Irritable bowel syndrome (IBS) is a chronic gastrointestinal (GI) disorder that reportedly affects 5% to 20% of the world population. The etiology of IBS is not completely understood, but diet appears to play an important role in its pathophysiology. Asian diets differ considerably from those in Western countries, which might explain differences in the prevalence, sex, and clinical presentation seen between patients with IBS in Asian and Western countries. Dietary regimes such as a low-fermentable oligo-, di-, monosaccharides, and polyols (FODMAP) diet and the modified National Institute for Health and Care Excellence (NICE) diet improve both symptoms and the quality of life in a considerable proportion of IBS patients. It has been speculated that diet is a prebiotic for the intestinal microbiota and favors the growth of certain bacteria. These bacteria ferment the dietary components, and the products of fermentation act upon intestinal stem cells to influence their differentiation into enteroendocrine cells. The resulting low density of enteroendocrine cells accompanied by low levels of certain hormones gives rise to intestinal dysmotility, visceral hypersensitivity, and abnormal secretion. This hypothesis is supported by the finding that changing to a low-FODMAP diet restores the density of GI cells to the levels in healthy subjects. These changes in gut endocrine cells caused by low-FODMAP diet are also accompanied by improvements in symptoms and the quality of life.

## Introduction

Irritable bowel syndrome (IBS) is a chronic functional disorder. Although the etiology of IBS is unknown, several factors seem to play a role in its pathophysiology; these include genetic factors, diet, intestinal microbiota, low-grade inflammation, and abnormalities of the gastrointestinal (GI) endocrine cells [1].

IBS patients suffer from intermittent lower abdominal pain, altered bowel habits, and abdominal bloating/distension [2]. IBS is mainly diagnosed based on an assessment of the symptoms described by the Rome criteria [3]. Based on the stool pattern, IBS patients are divided into four subtypes: diarrhea-predominant, constipation-predominant, mixed diarrhea and constipation, and unclassified [4, 5]. IBS patients are further divided into two subsets: sporadic (non-specific) and post-infection [6]. Sporadic IBS includes patients who have had symptoms that are not associated with GI infections. Post-infection IBS occurs in otherwise healthy subjects as the sudden onset of IBS symptoms after an episode of gastroenteritis [6], and reportedly constitutes 6% to 17% of patients with IBS [7].

IBS patients visit physicians more often than patients with diabetes mellitus, hypertension, or asthma; 12% to 14% of primary care visits and 28% of gastroenterologist referral are due to IBS [8–10]. The onset of IBS is usually in young age (< 45 years) during the most active phase of their lives [11, 12]. There is a sex difference in the prevalence of IBS, with a female:male ratio of 2:1 [11]. IBS reduces the quality of life to the same degree as other chronic diseases such as diabetes mellitus and kidney failure [13].

The present review discusses the role of diet in the pathophysiology of IBS, the use of diet in its management, and the possible mechanisms behind the effects of diet on IBS.

## Role of diet in IBS

IBS patients associate their symptom with food intake, especially specific food items such as wheat products, milk and its products, cabbage, onion, hot spices, and fried and smoked foodstuffs [14, 15]. Despite IBS patients avoiding certain food items, diet surveys have not found any differences in intake between IBS patients and community controls [16]. Furthermore, there is no difference between IBS patients and the background population in the intake of calories, carbohydrates, proteins, or fats [15]. However, the diets of IBS patients tend to be low in calcium, magnesium, phosphorus, vitamin B_2_, and vitamin A [15].

While the prevalence of IBS in Asia is 5% to 9% [17–28], it is 10% to 20% in Western countries (USA and Europe) [14–25]. Furthermore, no female predominance was found in Asian IBS patients [2], in contrast toWestern IBS patients [22–25]. The clinical presentation of IBS differs between Asian and Western patients. Abdominal bloating is a more-common complaint than pain in Asians, and this abdominal pain is localized to the upper abdomen rather than in the lower abdomen in Asian patients. Moreover, alteration in bowel habits is much less prominent in Asian IBS patients than in their Western counterparts [2–13].

The Western diet is rich in fats and beef protein and low in carbohydrates and fiber, whereas the Asian diet is rich in carbohydrates and fiber, and low in fats and meat protein [29]. Asian meals consist mainly of rice, vegetables, fish, eggs, poultry, pork, vegetable oil, and spices [29]. Rice is absorbed in the small intestine and produces negligible amounts of intestinal gas [29]. Furthermore, cellophane noodles made from mung bean flour, a traditional Asian food, produce much less intestinal gas than do wheat noodles [30]. These main differences between the diet in Asia and Western countries might at least partially explain the differences mentioned above between patients with IBS in Asian and Western countries, including in its prevalence, sex difference, and clinical presentation.

## Diet in the management of IBS

The intake of a low-fermentable oligo-, di-, monosaccharides, and polyols (FODMAP) diet reportedly improves both symptoms and the quality of life in 50% to 76% of IBS patients (Table 1) [16, 31–38]. Two recent systematic reviews of randomized trials produced significant evidence for the short-term benefits of a low-FODMAP diet on GI symptoms and quality of life in patients with IBS [39, 40]. However, the studies included in these reviews were of low quality, due to methodological heterogeneity and a high risk of bias, which commonly occurs in studies of dietary treatments. However, a low-FODMAP diet requires intensive meal-planning, is expensive and difficult to maintain over a long period, and can result in negative changes in the intestinal microbiota [35, 41–43]. Furthermore, consuming a low-FODMAP diet for a long time may result in deficiencies in vitamins, minerals, and naturally occurring antioxidants [42, 43]. The benefits of low-FODMAP diet have been demonstrated in all IBS subtypes, but IBS symptoms were found to improve less in patients with constipation-predominant IBS in two studies [36, 44].

**Table 1:** Summary of randomized trials of a low-FODMAP diet compared with the usual diet or common dietary advice in the management of patients with irritable bowel syndrome

Study	Participants	Intervention	Control	Study duration, design	Results
Staudacher et al., 2011 [34]	*N* = 41; unknown IBS subtype	Low-FODMAP	Usual diet	4 weeks; advice	• Significantly greater proportion of patients with adequate symptom control (68% vs. 23%, low-FODMAP vs. control) • Significantly lower concentration and proportion of bifidobacteria in low-FODMAP group
Böhn et al., 2015 [36]	*N* = 38; moderate to severe symptoms; IBS-C (*n* = 22), IBS-D (*n* = 18), IBS-M (*n* = 35)	Low-FODMAP	Traditional IBS diet (greater emphasis on how and when to eat than on what foods to ingest)	4 weeks; advice	• Overall symptoms (IBS-SSS score) significantly reduced after treatment in both groups, with no significant intergroup difference
Eswaran et al., 2016 [35]	*N* = 92; IBS-D	Low-FODMAP	Modified NICE	4 weeks; advice	• Significantly greater proportion of patients with symptom relief ≥ 50% (52% vs. 41%, low-FODMAP vs. control) • Significantly improved abdominal pain, bloating, stool consistency, frequency, and urgency • Significantly improved IBS-QoL score, anxiety, and activity impairment
Zahedi et al., 2018 [38]	*N* = 101; IBS-D; 78% with moderate to severe symptoms	Low-FODMAP	General advice (British Dietitian Association recommendation 2016)	6 weeks; advice	• Significantly improved scores overall and for the individual items on IBS-SSS, stool consistency, and stool frequency
Patcharatrakul et al., 2019 [37]	*N* = 62; IBS-C (*n* = 20), other non IBS-C subtypes (*n* = 42)	SILFD	Brief advice on commonly recommended IBS diet	4 weeks; advice	• Significantly greater proportion of patients with ≥ 30% decrease in average of daily worst abdominal pain/discomfort (60% vs. 28%, structural vs. brief advice) • Significant lower post-prandial hydrogen gas production in SILFD compared with brief advice

*IBS-D* diarrhea-predominant IBS, *IBS-C* constipation-predominant, *IBS-M* mixed diarrhea and constipation IBS, *IBS-SSS* *IBS* symptom severity score, *IBS-QoL*, IBS-quality of life; *SILFD* structural individual low-fermentable oligo- di- monosaccharide and polyol (FODMAP) advice; *NICE* National Institute for Health and Care Excellence

The modified National Institute for Health and Care Excellence (NICE) diet has the same effect as a low-FODMAP diet in around half (46% to 54%) of IBS patients, but it is easy to maintain and does not have the hazards seen with a low-FODMAP diet [35, 36]. The modified NICE diet is the first diet recommended for IBS patients by the British Dietetic Association [15, 16, 45, 46]. In the modified NICE diet, patients are advised to consume regular meals; to replace wheat products with spelt products; to reduce the intakes of fatty food, onions, cabbage, and beans; to avoid carbonated beverages, and sweeteners whose names end with “-ol”; and to regularly consume psyllium husk fibers. The British Dietetic Association also recommends reducing the intakes of coffee, spicy foods, and alcohol [15, 16].

## Role of diet in the pathophysiology of IBS

Diet induces IBS symptoms via several mechanisms, including intestinal distention, direct chemical stimulation of the epithelial cells lining the intestinal lumen, and altered intestinal microbiota. There is no evidence that IBS patients suffer from a food allergy mediated by immunoglobulin E [47–55]. It has also been documented that food intolerance does not occur in IBS, which is a non-toxic, non-immune-mediated reaction to bioactive chemicals in food [2, 55].

**Fig. 1:**
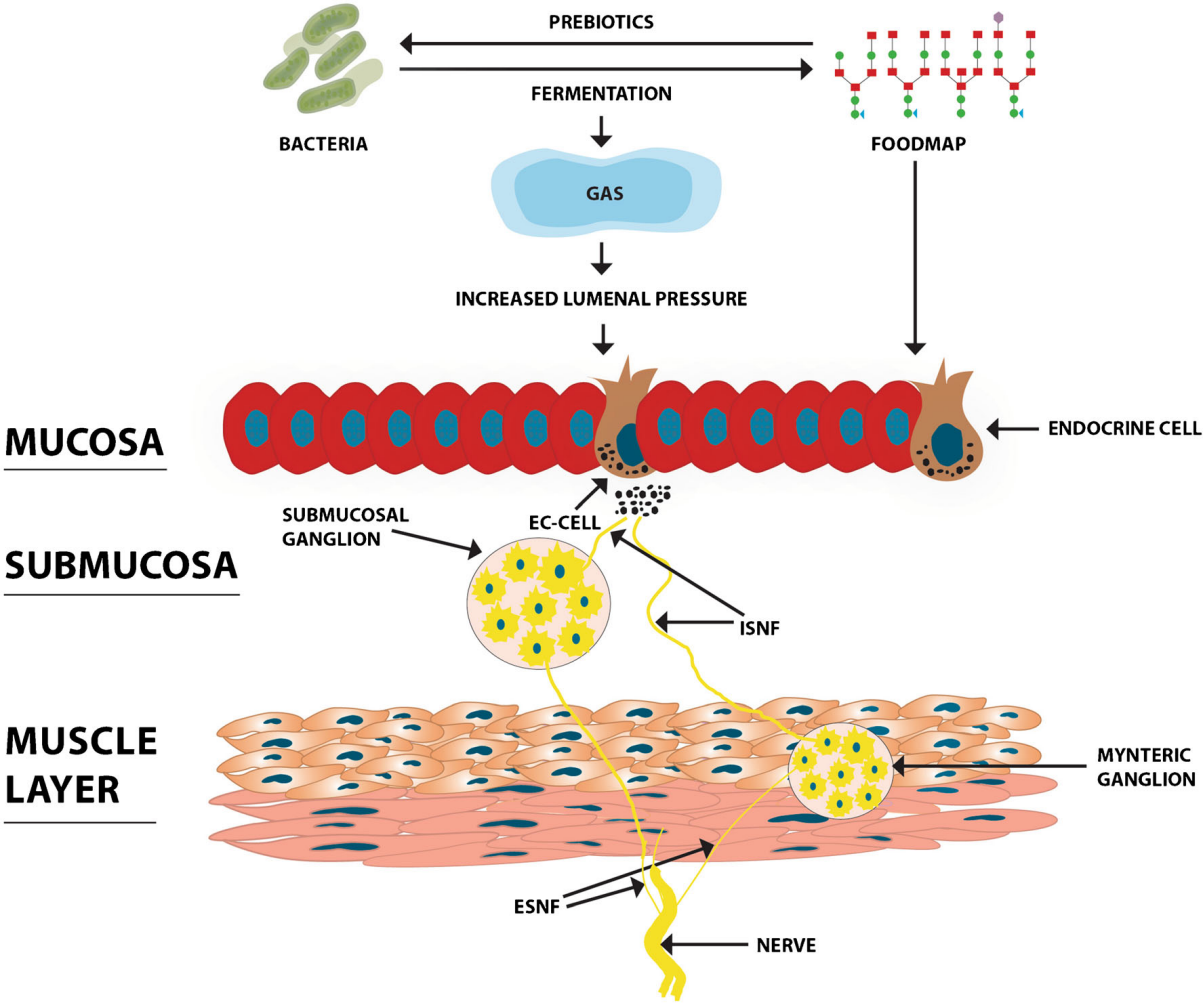
Schematic illustration of the possible mechanisms by which fermentable oligo- di- monosaccharide and polyol (FODMAP) can trigger IBS symptoms. Upon reaching the large intestine, FODMAP increases the osmotic pressure and act as prebiotics for gas-producing *Clostridium* bacteria, inducing an increase in gas production. The production of gas increases the luminal pressure and stimulates the release of serotonin from enterochromaffin (EC) cells. Serotonin acts on the intrinsic sensory nerve fibers (ISNF) of the submucosal and myenteric ganglia, which in turn convey the activation via the extrinsic sensory nerve fibers (ESNF) to the central nervous system. They also act indirectly on the enteroendocrine cells that regulate gastrointestinal sensation, motility, secretion, and absorption. Reproduced from El-Salhy and Gundersen [31] with permission from the authors and publisher

The intestine can be distended by the amount/volume of ingested food, fluid secretion induced by ingested food, and gas production caused by intestinal bacteria fermenting food. GI wall distention is well known to induce symptoms of fullness, distention, and abdominal pain [56]. Early symptoms can also develop as a direct effect of food components such as chili, since this contains capsaicin that induces abdominal burning and pain [57]. The modulation of GI motility and sensation via neurohormonal mechanisms such as fatty food was found to induce gut hypersensitivity and delay the GI transit of gas and the contents [14, 58]. There are several possible mechanisms by which a FODMAP-containing diet can trigger IBS symptoms (Fig. 1), such as by increasing the osmotic pressure in the large intestine, acting as prebiotics for gas-producing *Clostridium* bacteria, providing a substrate for bacterial fermentation causing abdominal distension and pain/discomfort, and interacting with the enteroendocrine cells that regulate GI sensation, motility, secretion, and absorption.

A small proportion of IBS patients may have undiagnosed celiac disease and may develop GI symptoms after ingesting foodstuffs that contain wheat or gluten. A meta-analysis suggested that the prevalence of biopsy-demonstrated celiac disease in cases meeting diagnostic criteria for IBS was 4%, which was fourfold higher than that in controls without IBS [59]. Celiac disease was diagnosed by serology and biopsy in 1% of Chinese patients with diarrhea-predominant IBS according to the Rome-III criteria [60].

Non-celiac gluten sensitivity (NCGS; better termed non-celiac wheat sensitivity) is defined as having GI and extra-GI IBS-like symptoms without celiac disease or wheat allergy, but the symptoms are relieved by a gluten-free diet (GFD) and relapse upon a gluten challenge [61–66]. The prevalence of NCGS has been reported as 0.55% to 6% of the US population [63, 67]. A randomized, double-blind, cross-over, food challenge study using gluten protein and control protein in IBS patients who were already consuming a low-FODMAP diet demonstrated that gluten protein produced symptoms similar to whey protein (as a control) [68]. Furthermore, 24% of those who believed that they had NCGS experienced uncontrolled symptoms despite consuming a GFD [69]. This suggests that the aggravation of GI symptoms in IBS patients who consume a gluten diet is the result of the carbohydrate content (fructans and galactans) of wheat rather than gluten.

As mentioned above, diet, the intestinal bacterial flora, and abnormalities of the GI endocrine cells play an important role in the pathophysiology of IBS [1, 70–72]. Moreover, these factors seem to interact with each other [13].

**Fig. 2:**
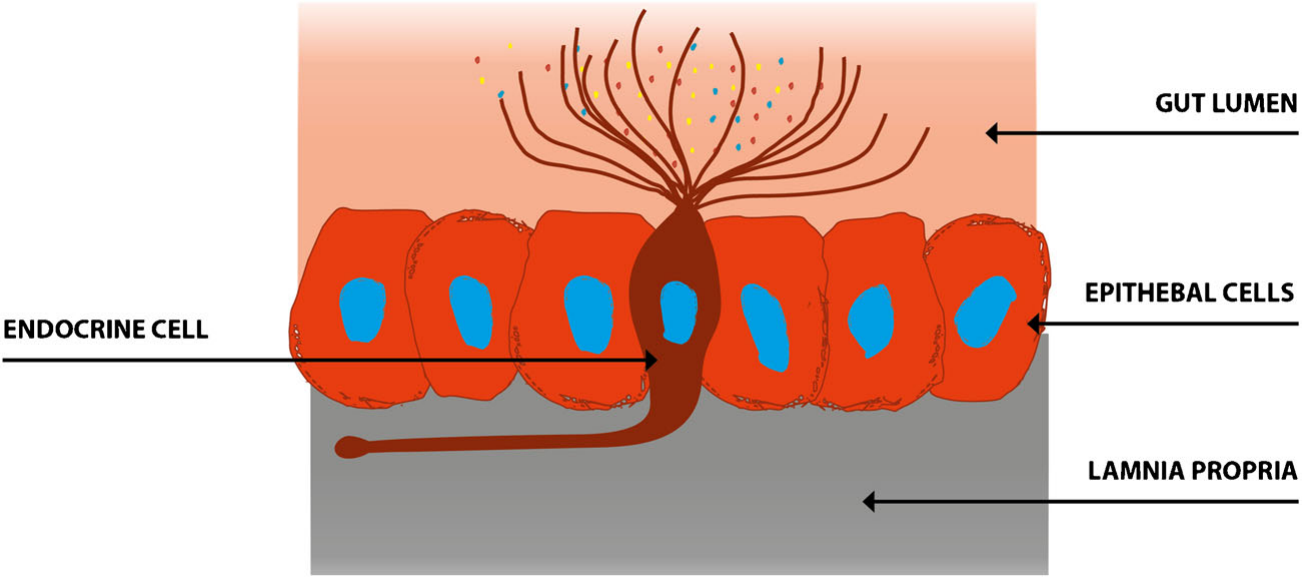
The gut endocrine cells have specialized microvilli that project into the lumen and act as sensors for the luminal contents (mostly for nutrients). These respond to the luminal contents by releasing hormones into the lamina propria. These hormones act locally on the nearby structures (paracrine mode of action) or enter the bloodstream and act on distant structures (endocrine mode of action). Reproduced from El-Salhy et al. [13] with permission from the authors and publisher

The enteroendocrine cells are scattered between the epithelial cells facing the gut lumen [6, 73]. The enteroendocrine cells have sensors in the form of microvilli protruding into the lumen, and these sense the gut contents (mostly nutrients) and respond to them by releasing hormones into the lamina propria (Fig. 2) [74–86]. The hormones released from the enteroendocrine cells vary with the gut intraluminal contents and the proportions of carbohydrates, proteins, and fats [6, 73]. These released hormones regulate visceral sensation, motility, secretion, absorption, the local immune defense system, cell proliferation, and appetite [2, 73, 87–89]. Patients with IBS exhibit gut dysmotility, visceral hypersensitivity, and abnormal secretion [90]. The densities of enteroendocrine cells are generally lower in IBS patients than in healthy subjects [91], and these abnormalities appear to play a major role in the pathophysiology of IBS [90, 92]. It is particularly interesting that the density of enteroendocrine cells in the intestine did not differ between Thai IBS patients and healthy controls, nor did the densities of stem cells or progenitors for enteroendocrine cells [93, 94].

**Fig. 3:**
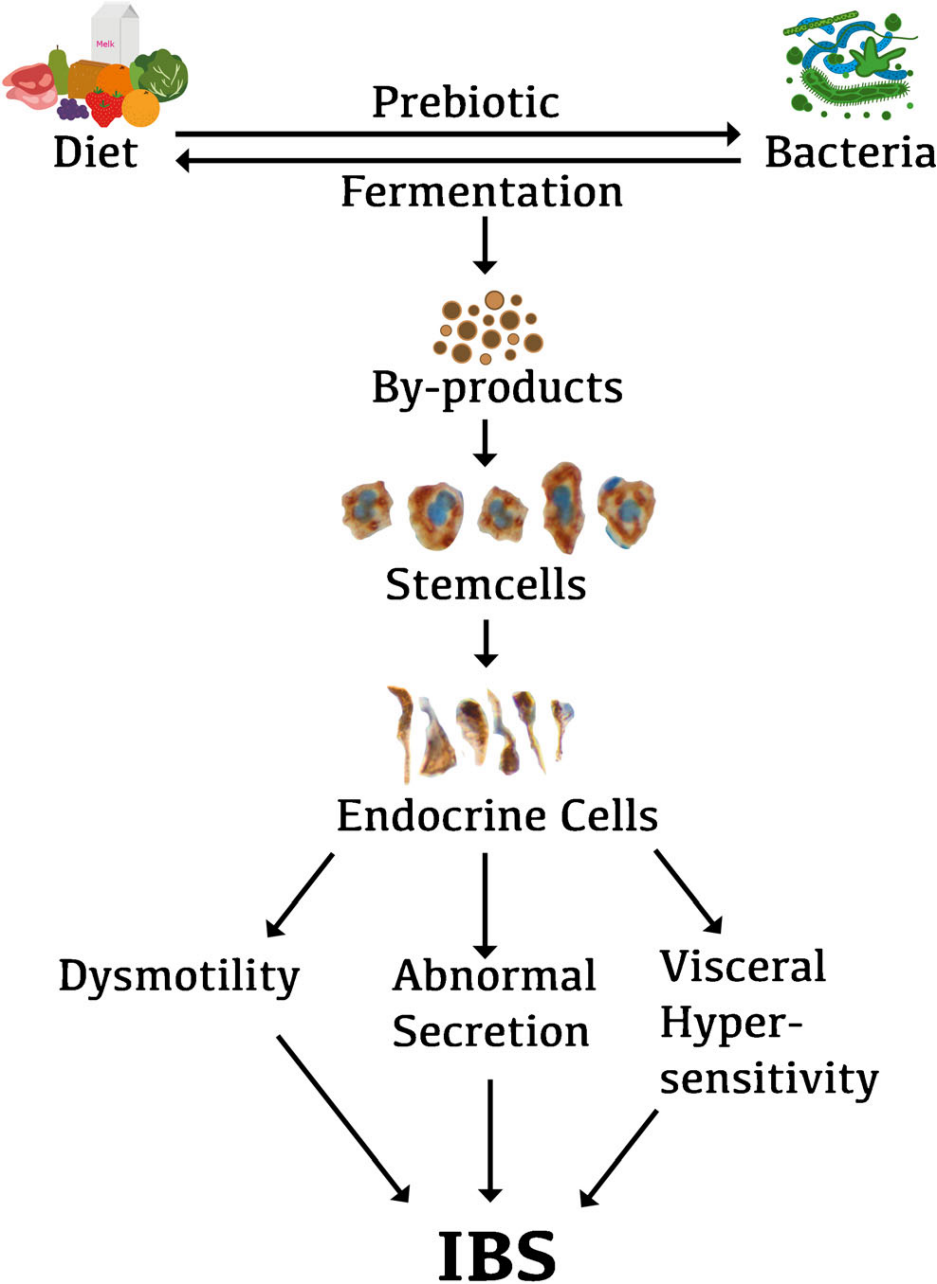
Schematic illustration of the possible role of interactions between diet, gut microbiota, and gut endocrine cells in the pathophysiology of irritable bowel syndrome (IBS). The diet that we consume acts as a prebiotic that favors the growth of certain types of bacteria. These bacteria can in turn ferment the diet, resulting in by-products that may act on the stem cells in a way that reduces their number. This in turn would result in a low density of gut endocrine cells, which gives rise to the gut dysmotility, visceral hypersensitivity, and abnormal gut secretion seen in IBS patients. Reproduced from El-Salhy et al. [13] with permission from the authors and publisher

It is hypothesized that diet as a prebiotic can favor the growth of certain bacteria. The fermentation products of such a diet produced by these bacteria act on intestinal stem cells to cause a low differentiation into endocrine cells. The resulting low density of enteroendocrine cells and the subsequent low levels of certain hormones give rise to intestinal dysmotility, visceral hypersensitivity, and abnormal secretion (Fig. 3) [13].

**Fig. 4:**
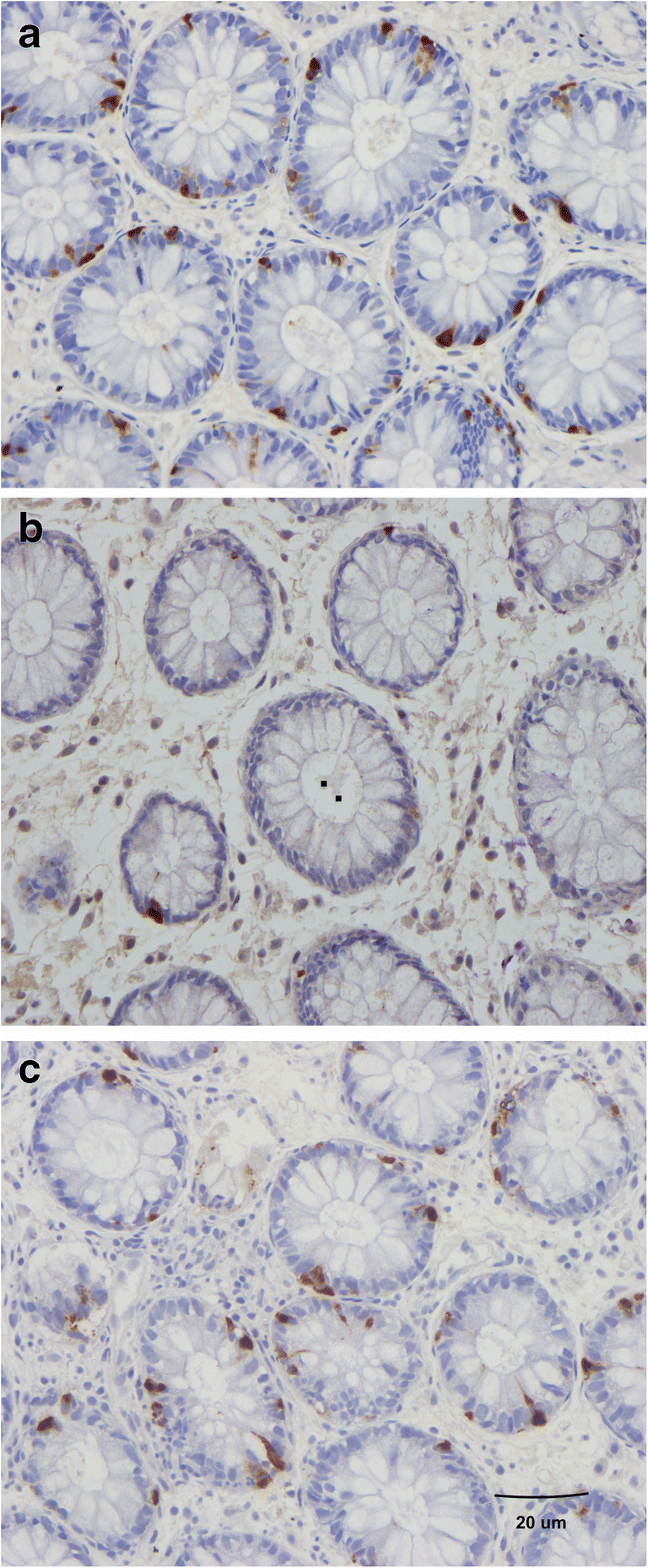
Colonic chromogranin A cells in a healthy subject (**a**), in a patient with irritable bowel syndrome (IBS) (**b**), and in a patient with IBS after 3 months on a low-FODMAP diet (**c**). Chromogranin A is a common marker for enteroendocrine cells

**Fig. 5:**
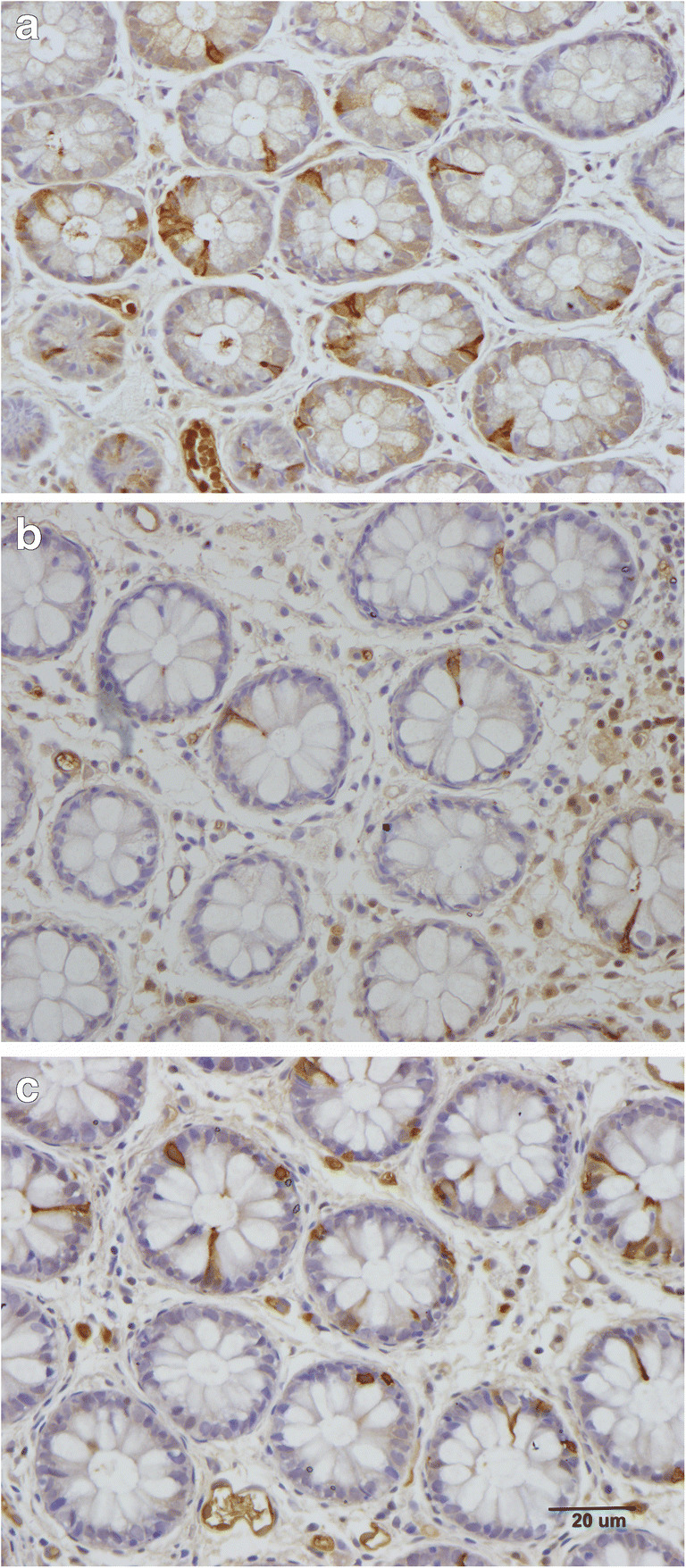
Peptide YY (PYY) cells in the colon of a healthy control (**a**), in a patient with irritable bowel syndrome (IBS) (**b**), and in a patient with IBS after 3 months on a low-FODMAP diet (**c**). PYY regulates the intestinal motility, which is a major mediator of the ilial break, as well as the absorption of water and electrolytes in the colon

This hypothesis is supported by the finding that changing from the common Norwegian diet of IBS patients to a low-FODMAP diet restored the density of GI cells to the levels in healthy subjects (Figs. 4 and 5) [unpublished data, 95–102]. In addition, Thai IBS patients consuming different favorable diets for IBS did not show abnormalities in the enteroendocrine cells [93, 94]. Moreover, fecal microbiota transplantation changes the densities of intestinal endocrine cells in patients with IBS towards those seen in healthy subjects [103]. These changes in gut endocrine cells caused by a low-FODMAP diet and fecal microbiota transplantation were accompanied by improvements in symptoms and the quality of life [99].

## Conclusion

Diet plays an important role in the pathophysiology of IBS by interacting with other factors that affect the pathophysiology, namely the intestinal bacterial flora and enteroendocrine cells. Differences between Asian and Western diets may account for the differences between patients with IBS in Asia and Western countries, including in the prevalence, sex, and clinical presentation.
